# Attitudes toward Regulations of Reproductive Care in the European Union: A Comparison between Travellers for Cross-Border Reproductive Care and Citizens of the Local Country

**Published:** 2016-09

**Authors:** R Hertz, MK Nelson, J Suñol

**Affiliations:** Wellesley College, Departments of Sociology and Women’s and Gender Studies, Wellesley, MA 02481, USA; Middlebury College, Department of Sociology and Anthropology, Middlebury, VT, O5753, USA; Instituto Marquès, Medicina reproductiva, Barcelona, Spain

**Keywords:** European Union, gamete donation, political attitudes, cross-border reproductive care

## Abstract

This paper compares two populations with respect to attitudes toward the regulation of reproductive care by the European Union. The two populations are 252 individuals who crossed a national border to receive treatment at an independent clinic in Spain and 45 Spanish citizens who received treatment in their home country. Online surveys were sent to former patients (from many different countries) of a private Spanish clinic. By comparing those who engaged in cross-border reproductive care (CBRC) with those who did not, we examined attitudes toward whether or not the EU should extend to all clients in all countries the type of services the clinic provided. These services included access to anonymous donors and conception via egg or embryo donation. We found that those who travelled abroad were less in favor of EU expanding regulation for the type of services they received than were those in Spain. This study is unusual in focusing on political attitudes rather than the nature of the experience and consequences of cross-border reproductive care. We suggest that individuals who engage in CBRC might be reluctant to see the EU extend reproductive care broadly because debates within both the EU and their home countries could result in the elimination of options that are now available through travel. We suggest that individuals from countries that are popular destinations for CBRC like Spain might want to extend EU reproductive care more broadly so as to reduce the pressure on the medical services in their own country. We suggest directions for further research.

## Introduction

At the core of the European Union is consensus surrounding economic concerns. The EU founding treaty also includes a statement about improving public health and preventing human illness (“EUROPA - Topics of the European Union - Health,” n.d.) and unifying health policies do occur when people cross borders for temporary stays (“EUROPA - Topics of the European Union - Health,” n.d.). Yet, while countries may prohibit certain medical treatments such as particular kinds of reproductive services at home, freedom of movement between countries allows individuals to access these treatments elsewhere. A task force established by the European Society of Human Reproduction and Embryology (ESHRE) (Pennings et al., 2008) recommended that countries establish less restrictive guidelines to reduce the number of patients that have to travel abroad for treatment, and that wherever treatment is provided, safety and quality of treatment should be guaranteed (Shenfield et al., 2011). Similarly, the Ethics Committee of the American Society for Reproductive Medicine (2013 p.649) concluded that the physicians who treat ART patients from abroad should be held to the same standards of care for all patients within their jurisdiction. It also concluded that the delivery of care to patients from other countries does not require extra explanation: that care “does not invoke a duty to inform or warn patients about the potential legal or practical hazards that may accompany such care.” Blyth’s (2010) study of the experiences of individuals who travel across borders for reproductive care shows that individuals often do the bulk of the research themselves (much of it on the Internet) about available procedures and available clinics. He thus emphasizes (as did the ESHRE task force) “the need for accessible, accurate, and reliable information to help ensure safe and high quality care.” Others also call for more information to be made available to patients who cross borders for reproductive care and for protections to be in place (Collins and Cook, 2010; Culley et al., 2011; Karkanaki et al., 2012). Still, even though there is great acknowledgement that people move between countries for reproductive health care and that this movement can be problematic for social, emotional and medical wellbeing (Culley et al., 2011; Hudson et al., 2011; Lui et al., 2014), the EU has made no coordinated efforts to establish consistent services or consistent policies on the use of gametes or surrogates.

The complexity of reproductive regulations across Europe and their implication for reasons for travel have been the focus of much scholarly writing on cross-border reproductive care (CBRC). Studies show that those who travel often do so for multiple reasons (Shenfield et al., 2010; Culley et al., 2011; Pennings, 2009). Some seek to evade the legal restrictions in their home country because of bans on access to reproductive services for certain groups such as single women or the members of lesbian couples (Präg and Mills, 2015), bans on specific assisted reproductive treatments (Inhorn, 2015) and/or bans on particular types of gamete use such as donated eggs and/or embryos (Bergmann, 2011; Glennon, 2016). Others travel to avoid the high costs of services, long wait times, or what is perceived to be a low-quality of care or an inadequate level of technological expertise at home (Gomez and de La Rochebrochard, 2013; Van Hoof et al., 2015). Finally, some might be travelling to maintain secrecy about their use of donor gametes, particularly since attitudes about disclosure are far from uniform (Laruelle et al., 2011). Travellers might also want to maintain secrecy to prevent the stigma associated with reliance on donor gametes (Inhorn and Gürtin, 2011) or might worry about prosecution for reliance on procedures prohibited in their own country (Van Hoof and Pennings, 2011).

Some of this travel takes a particular form, involving specific departure countries and specific destination countries. Departure countries are also the individual’s home country whereas travellers are treated in destination countries for CBRC. For example, among Europeans, Krolokke (2014) notes the specifics of reproductive flows. She points out that Italians, Swedes, and Norwegians remain in their home countries and receive sperm from anonymous donors in Denmark, while Danes, Norwegians and Germans travel to Spain, Greece, the Ukraine, or the Czech Republic for eggs from anonymous donors. Pennings (2009) documents a different flow of patients, noting that the majority of foreign patients in Belgium were French women for sperm donation travelling to circumvent French law. All such movements reflect the particular legal regulations of destination countries and the nature of the restrictions at home. Regardless of the particular motivations or destinations, travellers often report feeling “exiled” from their home countries because the restrictive politics at home ignore their reproductive needs (Inhorn and Patrizio, 2009).

Spain, which is the site for this study, is an important destination for CBRC because it has limited restrictions, short waiting lists and a sufficient number of available gamete donors to meet the demand (Shenfield et al., 2010; Culley et al., 2011; Gomez and de La Rochebrochard, 2013; Kroløkke, 2014). Spanish law also protects the lifelong anonymity of all medical donors (Baccino et al., 2014).

Some studies look at the effects of CBRC not just from the perspective of the immediate parties involved, but also from the perspective of the European context and its varying legal restrictions that constitute all departure and destination countries. Van Beers (2015 p.103) argues against those who suggest that the ease with which the laws in an individual country can be evaded makes a mockery of those laws. He insists that existing laws continue to “have an important communicative, expressive and anthropological meaning and function, which surpass these laws’ practical effectiveness.” In a somewhat different analysis Storrow (2010 p. 2939) argues against CBRC because of its effects on the countries from which people travel. Noting that “the opportunity for patients to go abroad for treatment tempers organized resistance to the law and allows government to pass stricter regulations than it otherwise might,” he suggests that those who travel abroad will no longer speak out in opposition to the laws that their countries have – the very laws that would have prevented them from receiving treatment in their own countries in the first place. Pennings (2009) similarly views CBRC as a “safety valve” that takes the pressure off the restrictive legislation in a particular country. Storrow (2010) goes one step further when he suggests that there might be effects on the population in destination countries both in the possible exploitation of those providing donor gametes and in raising the costs (and otherwise distorting) the medical care provided in the destination country. Storrow argues that it is not necessarily in the interest of the destination country to provide all services requested by cross- border travellers.

Storrow’s analysis raises the question of the attitudes of those who engage in CBRC and, more particularly, whether those who engage in CBRC are more or less likely than those who find the same arrangements in their own countries to prefer that the EU create a uniform body of laws governing access to reproductive technologies rather than the varied regulations that now exist. We can compare the attitudes of two groups toward the issue of EU legislation: border crossers to Spain and Spanish citizens who receive the same (or similar) treatment in their home country and thus did not have to travel to receive treatment. This analysis can help us understand both the interests and concerns of those who cross borders in search of reproductive care and Spanish citizens who receive care in Spain.

## Materials and Methods

### Data Collection

The data came from an online survey of former patients who were successfully treated by a fertility clinic in Spain and who came from many countries. Clinic personnel sent an email invitation to all former patients (N=1296) who conceived and gave birth to children through donor gametes or embryos in the previous five years. The email offered links to the survey with four language options: English, Spanish, French and German. The survey options were translated from an original survey written in English to the other three languages by two individuals who are U.S.-based translators. To ensure that the survey translations, especially the technical language of gametes, were accurate the clinic’s personnel commented on each translation. The online surveys were translated back to English and they were also checked to make sure the questions were identically formatted. The survey was online from November 15, 2015 to January 15, 2016. After the survey closed the data from the four sets of respondents were combined into one data set. Ethical approval for this study was obtained from the Institutional Review Boards of the U.S. institutions represented by the first two authors. The overall response rate was 23%.

The survey collected background information about the respondents including the reasons for doing so for those who had crossed a border to be treated in Spain. Questions about attitudes toward EU regulation and questions concerning to whom parents disclosed their reliance on donor gametes were pretested by the authors prior to administering the survey. Some of the questions had previously been used in earlier studies of US donor-conceived families (Freeman et al., 2009; Sawyer et al., 2013; Hertz et al., 2016).

The key attitudinal question having to do with EU regulations consisted of a series of statements each of which was scored on a five point Likert scale from “strongly agree” to “strongly disagree.” We analysed these data using the nominal categories as opposed to means because to do the latter would make assumptions about the data; we then apply the appropriate tests of significance for categorical data. We also analysed the data by separating “strongly agree” from the other categories. We do not assume the ordinal measure is a continuous scale. “Strongly agree” respondents held unambiguous attitudes.

For a question about political attitudes in general respondents were asked where they would place themselves on a 10-point scale from 1 (Left) to 10 (Right); for purposes of analysis we divide the respondents into three groups with 1 to 4 being “Left,” 5 being “Middle,” and 6 to 10 being “Right.” For purposes of analysis we used two variables regarding the respondents’ age; the respondent’s present age and the respondent’s age at the time of the conception of their first donor-conceived (DC) child. We also asked respondents to report on their own estimate of social class and we divided them into two groups: those saying middle class or higher and those saying less than middle class (i.e., working class and lower class). Finally, we divided educational level into two groups: those with at least a university (BA) degree and those with less education.

### Analysis

We compared the two groups in terms of background variables (including political attitudes) (Table IA and IB). For the border crossers we explored their reasons for doing so (Table II). We also compared the attitudes of the border crossers with the non-border crossers with respect to a variety of statements about EU regulations (Table III). In order to better understand the statistically significant differences (as measured by Chi-square Tests) between border crossers and non- border crossers with respect to attitudes toward EU regulations, we considered whether the background variables on which the two groups differed had statistically significant associations with statements about the EU (Table IV). For this part of the analysis we only examined a variable if it was shown both to be different for the CBRC and Spain and to have its own association to attitudes toward the EU. For example, the CBRC and Spain populations differed in terms of ethnicity (with fewer of the Spain respondents identifying as White/Caucasian). In addition, as shown in Table IV, ethnic identification had a statistically significant association to one of the EU questions (whether all people should be treated for reproductive care). Therefore, we examined the CBRC/Spain difference in the proportions giving that response among both the non-Caucasian and the Caucasian populations for that question.

**Table IA:** — Demographic differences between CBRC respondents and Spain respondents

	CBRC	SPAIN	ALL	Chi-square Test p-value
Percent Catholic while growing up	34	71	39	0.00
*N=*	*262*	*45*	*297*	
Percent Catholic now	24	40	27	0.02
*N=*	*252*	*45*	*297*	
Percent Female	87	98	89	0.05
*N=*	*249*	*45*	*294*	
Percent Heterosexual	96	96	96	
*N=*	*250*	*45*	*295*	
Percent Caucasian	94	73	91	0.00
*N=*	*252*	*45*	*297*	
Percent education above Secondary School	74	61	72	0.05
*N=*	*226*	*43*	*269*	
Percent Middle or Upper Social Class	83	67	81	0.02
*N=*	*243*	*43*	*286*	
Percent Age 45 or older	60	27	55	0.00
*N=*	*247*	*45*	*292*	
Percent Age 40 or Older at conception of First Donor-Conceived Child	80	44	74	0.00
*N=*	*234*	*39*	*273*	
Percent Partnered	92	88	91	
*N=*	*213*	*41*	*254*	
Percent who disclosed donor conception to child	25	3	22	0.00
*N=*	*239*	*39*	*278*	
Percent who has told No one about donor conception	5	15	7	0.04
*N=*	*239*	*39*	*278*	

**Table IB:** — Political differences between CBRC respondents and Spain respondents

	CBRC	SPAIN	ALL
Right	36%	27%	35%
Moderate	27%	19%	26%
Left	37%	56%	40%
Total	100%	100%	100%
N=	250	43	293

**Table II:** — Reasons for CBRC by Home Country (Among those with >10 Respondents) (Percent giving each reason)

	England	France	Germany	Ireland	Scotland	Australia	Norway	Total
No Access to gametes in home country	4	50	17	19	13	21	36	14
Not enough gametes in home country	59	29	4	19	40	50	0	34
Wanted anonymous donor	45	7	4	12	33	36	9	26
Wanted gametes not allowed in home country	3	0	91	19	0	14	64	16
Could be treated more quickly	69	36	17	50	47	64	18	45
Tried at home and was unsuc- cessful	33	57	30	46	20	50	36	30
Partner tired at home and was unsuccessful	8	7	0	12	7	0	9	5
Procedures less costly than in home country	10	0	0	23	0	14	0	7
Gametes less costly than in home country	4	0	0	4	0	0	0	2
N=	117	14	23	26	15	14	11	297

**Table III:** — Attitudes toward EU regulation (Percentage (%) who “Strongly Agree”)

The EU and all countries around the world should have uniform laws which:	CBRC (N=252)	SPAIN (N=45)	Chi-square Test p-value
Allow all people to be treated for infertility	52	96	0.00
Allow all people to use donated gametes and embryos	47	82	0.00
Allow all people to use surrogates	32	58	0.00
Find a quicker way to offer services to people with infertility problems	49	84	0.00
Reduce the costs of assisted reproductive technologies and the use of donors	50	87	0.00
Require anonymity of donors	25	71	0.00
Give people who need donor assistance to create their families the ability to make their own individual choices	43	80	0.00
The system in my country is fine the way it is	1	18	0.00

**Table IV:** — Demographic variables and attitudes toward EU regulation. Showing statistical significant responses only. (Percentage (%) who “Strongly Agree”)

The EU and all countries around the world should have uniform laws which:-	RELIGION GROWING UP			NOT CATHOLIC			CATHOLIC		
	Not Catholic (N = 180)	Catholic (N = 117)	Chi-square Test p-value	CBRC (n-167)	SPAIN (n=13)	Chi-square Test p-value	CBRC (N=85)	SPAIN (n=32)	Chi-square Test p-value
Allow all people to be treated for infertility	53	67	0.02	50	100	0.00	55	94	0.00
Find a quicker way to offer services to people with infertility problems	47	67	0.00	44	77	0.02	59	89	0.00
Reduce the costs of assisted reproductive technologies and the use of donors	49	65	0.01	46	92	0.00	58	84	0.01
The EU and all countries around the world should have uniform laws which:-	RELIGION IN CURRENT LIFE			NOT CATHOLIC			CATHOLIC		
	Not Catholic (N=218)	Catholic (N=79)	Chi- square Test p-value	CBRC (N=191)	SPAIN (N=27)	Chi- square Test p-value	CBRC (N=61)	SPAIN (N=18)	Chi- square Test p-value
Allow all people to be treated for infertility	55	67	0.04	49	96	0.00	59	94	0.00
Find a quicker way to offer services to people with infertility problems	5	63	0.05	47	85	0.00	57	83	0.04
The EU and all countries around the world should have uniform laws which:-	Age			Under 45			45 or Older		
	Under 45 (N=132)	45 or Older (N=160)	Chi- square Test p-value	CBRC (N=99)	SPAIN (N=33)	Chi- square Test p-value	CBRC (N=148)	SPAIN (N=12)	Chi- square Test p-value
Allow all people to be treated for infertility	66	53	0.02	57	94	0.00	49	100	0.05
Find a quicker way to offer services to people with infertility problems	63	48	0.04	55	88	0.00	46	75	0.05
The EU and all countries around the world should have uniform laws which:-	Age at which Had first DC Child			Under 40			40 or Older		
	Under 40 (N=70)	40 or Older (N=203)	Chi- square Test p-value	CBRC (N=48)	SPAIN (N=22)	Chi- square Test p-value	CBRC (N=186)	SPAIN (N=17)	Chi- square Test p-value
Allow all people to be treated for infertility	71	54	0.00	60	96	0.00	51	94	0.00
Find a quicker way to offer services to people with infertility problems	67	51	0.02	54	91	0.00	48	77	0.02
Reduce the costs of assisted reproductive technologies and the use of donors	66	52	0.03	56	86	0.01	49	82	0.01
The EU and all countries around the world should have uniform laws which:	Social Class			Lower or Working Class			Middle or Upper Class		
	Lower or Working Class (N=55)	Middle or Upper Class (N=231)	Chi- square Test p-value	CBRC (N=41)	SPAIN (N=14)	Chi- square Test p-value	CBRC (N=202)	SPAIN (N=29)	Chi- square Test p-value
Allow all people to use donated gametes and embryos	66	50	0.02	56	93	0.01	47	76	0.03
Give people who need donor assistance to create their families the ability to make their own individual choices	62	46	0.02	56	79	*	41	79	0.00
The system in my country is fine the way it is	9	2	0.03	2	29	0.01	1	14	0.00
Political Attitudes	NO Statistically Significant Relationships								
Education	NO Statistically Significant Relationships								
The EU and all countries around the world should have uniform laws which:-	Gender			Female					
	Female (n=262)	Male (N=32)	Chi- square Test p-value	CBRC (N=218)	Spain (N=44)	Chi- square Test p-value			
Allow all people to be treated for infertility	61	41	0.02	54	96	0.00			
Allow all people to use donated gametes and embryos	55	38	0.05	49	81	0.00			
Allow all people to use surrogates	38	19	0.02	34	57	0.04			
Find a quicker way to offer services to people with infertility problems	58	31	0.00	52	84	0.00			
Reduce the costs of assisted reproductive technologies and the use of donors	60	25	0.00	54	85	0.00			
Require anonymity of donors	34	16	0.02	27	71	0.00			
The EU and all countries around the world should have uniform laws which:	Ethnic Identity			Not Caucasian			Caucasian		
	Not White (N=26_	White (N=271)	Chi- square Test p-value	CBRC (N=14)	Spain (N=12)	Chi- square Test p-value	CBRC (N=238)	Spain (N=33)	Chi- square Test p-value
Allow all people to be treated for infertility	77	57	0.03	64	92	*	51	97	0.00
The EU and all countries around the world should have uniform laws which:-	Disclosed to Child			Disclosed to Child			Did Not Disclose to Child		
	Yes (N=61)	No (N=231)	Chi- square Test p-value	CBRC (N=6)	Spain (N=1)	Chi- square Test p-value	CBRC (N=188)	Spain (N=43)	Chi- square Test p-value
Allow all people to be treated for infertility	44	62	0.01	43	**	0.01	58	95	0.00
Allow all people to use donated gametes and embryos	41	55	0.04	42	**		48	84	0.00
Require anonymity of donors	16	36	0.00	17	**	0.01	28	72	0.00
The EU and all countries around the world should have uniform laws which:-	Disclosed to Someone			Disclosed to Someone			Disclosed to No One		
	Yes (N=278)	No (N=19)	Chi- square Test p-value	CBRC (N=239)	Spain (N=39)	Chi- square Test p-value	CBRC (N=13)	Spain (N=6)	Chi- square Test p-value
Require anonymity of donors	31	53	0.04	24	69	0	39	83	NS*
* Not significant at .05 level									

Finally, we also considered whether the different reasons for travel showed statistically significant associations with the different reactions to the statements about the EU. We examined whether the difference between the CBRC and Spain still persists among those with different reasons for travel to Spain (Table V). As was the case for the demographic variables, if the reason for travel has its own association to the EU attitudinal variables, we looked at the CBRC/Spain relationship within the reason for travel. Altogether there were three groups for comparison: Spanish citizens who do not travel (and therefore have no “reason” given), border crossers who gave a particular reason for travel and border crossers who did not give a reason for travel.

**Table V:** — Reasons for Travel and EU Attitudes (Showing Statistically Significant Responses Between those with Reason and Those without Reason Only)

	Couldn’t Get Access In Home Country					
	Not a Reason		Difference between CBRC and Spain	Reason for CB Travel	Difference Between Those with Reason and Those without	Difference Among all Three groups
The EU and all countries around the world should have uniform laws which:-	CBRC (N=211)	SPAIN (n=45)	Chi-square Test p-value	CRBC (N=41)	Chi-square Test p-value	Chi-square Test p-value
Allow all people to be treated for infertility	49	95	.00	66	0.03	0.00
Allow all people to use donated gametes and embryos	48	96	.00	70	.02	0.00
	Wanted Anonymous Donors					
	Not a Reason		Difference between CBRC and Spain	Reason for CB Travel	Difference Between Those with Reason and Those without	Difference Among all Three groups
The EU and all countries around the world should have uniform laws which:-	CBRC (N=175)	SPAIN (n=45)	Chi-square Test p-value	CRBC (N=77)	Chi-square Test p-value	Chi-square Test p-value
Require anonymity of donors	18	71	0.00	42	0.00	0.00
	Wanted Gametes Could Not Get in Home Country					
	Not a Reason		Difference between CBRC and Spain	Reason for CB Travel	Difference Between Those with Reason and Those without	Difference Among all Three groups
The EU and all countries around the world should have uniform laws which:-	CBRC (N=208)	SPAIN (n=45)	Chi-square Test p-value	CRBC (N=46	Chi-square Test p-value	Chi-square Test p-value
Allow all people to be treated for infertility	48	96	0.00	70	0.00	0.00
Allow all people to use donated gametes and embryos	43	82	*	65	0.01	0.00
	Tried in home country and Unsuccessful					
	Not a Reason		Difference between CBRC and Spain	Reason for CB Travel	Difference Between Those with Reason and Those without	Difference Among all Three groups
The EU and all countries around the world should have uniform laws which:-	CBRC (N=162)	SPAIN (n=45)	Chi-square Test p-value	CBRC (N=90)	Chi-square Test p-value	Chi-square Test p-value
Allow all people to use surrogates	27	58	*	41	0.01	0.00
Find a quicker way to offer services to people with infertility problems	44	84	0.00	59	0.02	0.00
Reduce the costs of assisted reproductive technologies and the use of donors	44	87	0.00	61	0.01	0.00
Require anonymity of donors	21	71	0.00	32	0.04	0.00
Give people who need donor assistance to create their families the ability to make their own individual choices	39	80	0.00	51	0.04	0.00
* Not significant at .05 level						

## Results

### Differences between Border Crossers and Spanish Residents

As shown in Table IA and IB there are differences between the two populations in religion, gender, ethnic identity, level of education completed, self-identified social class, current age, age at conception of first donor-conceived child and patterns of disclosure of reliance on donor gametes to children and others. Table IB shows that there are no differences between the two populations in political attitudes overall. As Table IA shows, there are also no differences between the two populations in percent who had a partner (whether married or not) at the time of conception, and sexual orientation at the time they responded to the survey.

The two populations are similar in the type of conception they experienced in the clinic. Over half of the respondents were women who received donor eggs and relied on their partner’s sperm; a third were women who received donor embryos, and 11% were men whose partners conceived with donor eggs and their own sperm (data not shown).

### Reasons for Border Crossing

Table II shows the reasons for traveling to Spain among the CBRC countries from which we received more than 10 responses as well as for the CBRC population as a whole. The respondents who crossed a national border to receive treatment in Spain came for a variety of reasons, which, to some extent, align with the country of origin. For example, those from England, where anonymity is banned, are more likely than those from other countries to say that they come to Spain because they want anonymity. Those coming from Germany and Norway wanted gametes not allowed in their home country. Among all the CBRC respondents, the most common reason for traveling was that one could be treated more quickly in Spain; the least common reason was that gametes were less costly than in the home country.

### Attitudes toward EU Legislation

In answer to a series of questions about whether the EU should create uniform regulations concerning reproductive care, there are significant differences between the two populations (Table III). In all cases, the respondents from Spain – respondents who had access precisely to what it was the other respondents were travelling to receive – were more likely than the border crossers to say that they strongly agreed with these extensions through EU legislation. The respondents from Spain believed that everyone in the European Union should have access to these medical services. They also were more likely to believe that their country was fine the way it was.

### Background Variables and Attitudes toward EU Regulation (Table IV)

**Religion:** The Spain respondents were more likely than those from the other countries to report being Catholic both while growing up and at the present time. Those who were Catholic while they were growing up were more likely to agree with the statements about everyone being treated, access to quicker services and access to less expensive services. Among both Catholics and non-Catholics the relationship between the CBRC respondents and the Spain respondents remained for each of these three issues.

The Spain respondents were also more likely to report being Catholic now and that variable is related independently to both the attitude toward access to treatment and access to quicker treatment. Once again, even within categories of Catholic or Not-Catholic, the difference between CBRC respondents and Spain respondents remained.

**Current Age:** The population of border crossers was older than the Spanish population and younger respondents were more likely to agree both with the idea of universal treatment and quicker access. Among both those who are younger and those who are older, the relationship between CBRC and Spain remains with respect to these two attitudinal variables.

**Age at birth of first DC child:** The Spanish population has also had its first DC child at a younger age and those who have had a DC child at a younger age are more likely to believe in universal treatment, quick treatment and reduced costs. Once again, the relationship between border crossing or not remained within categories of the age of the parent at the time of the first DC child.

**Social Class:** The population of respondents from Spain reported being of a lower social class than did those who are border crossers. Social class was associated with three of the items concerning EU regulation. In all three cases, those of a lower social class position were more likely to agree: broad use of donor gametes, people should be able to make their own choices, and whether or not their country’s legislation is fine the way it is. For the more privileged respondents, the difference between the border crossers and the Spanish population remained for all three of these variables. Among the less privileged respondents, the same difference remained but did not reach statistical significance with respect to the notion that people should make their own choices. The CBRC respondents were less in agreement than were the Spanish respondents.

**Political Attitudes:** The respondents who came to Spain from other countries were more likely to report being on the left side of a political spectrum but this difference was not statistically significant. Moreover, political attitudes were not independently related to attitudes toward the EU regulations and therefore we did not examine the difference between CBRC and Spain within different groupings of political attitudes.

**Education:** The Spanish population was less well educated than the CBRC population. However, education itself did not have a significant statistical relationship to attitudes toward EU legislation and therefore we did not examine the relationship between CBRC and Spain within different levels of education.

**Gender:** In the population of respondents only one man responded from Spain. All of the other male respondents were part of the CBRC pool. Gender was strongly related to attitudes toward the EU legislation. With only two exceptions, the men were more opposed to EU regularity than were women. The two exceptions were with respect to making one’s own choice and attitudes toward the current situation in one’s own country. Among women alone the difference between the Spain and CBRC populations remained and was statistically significant for each of the other six statements about the EU.

**Ethnic Identity:** Almost all of the CBRC respondents were White/Caucasian in comparison with less than three-quarters of those from Spain. Attitudes toward the EU legislation were essentially the same for both groups of respondents as differentiated by ethnic identity. However, more of the non-white population believed that the EU should provide access to fertility treatment for everyone who wanted it. Among the non-white respondents, the relationship between CBRC or not and EU attitudes was not statistically significant. Among the White respondents, however, the relationship remains: more Spain respondents believe that everyone should be treated.

**Disclosure:** Respondents from Spain were less likely than those who crossed borders to disclose to their children that they conceived with donor gametes. Although disclosure was more likely among those with older children, the same pattern prevailed within each age group of 0-2, 3-5 and 6 or more years of age. The CBRC respondents were less likely than those from Spain to say that they had told no one about their reliance on donor gametes. Disclosure to children was related to three EU attitudinal questions: whether all people should have access, whether all should be allowed to use donor gametes and whether anonymity should be required. Among those who had not disclosed donor conception to their child, the Spain respondents were more likely than CBRC respondents to agree with these statements. Disclosure to someone within one’s social circle was related to attitudes toward anonymity. Among those who had told someone, the relationship between CBRC and Spain respondents held; among those who had told no one, the relationship was in the same direction but did not achieve statistical significance probably because the numbers were too small in each of the two groups.

## Reasons for Border Crossing (Table V)

In most cases people gave more than one reason for cross-border travel for reproductive care and these reasons do not simply line up with the country of origin. We cannot simply say that people from one or another country come for a single reason. Some of these reasons are associated with attitudes towards the EU regulations. Among CBRC respondents alone, the difference between those who did and those who did not come to Spain for a particular reason was significant in ten distinct cases. In all except two instances we found additional statistically significant differences. Among those who did not come for a specific reason identified as being related to attitudes toward EU regulation, the CBRC and Spain respondents differed in all but two cases.

Those who said that they were border crossers because they were not allowed access to a particular form of fertility treatment in their own country were more likely to say that they believed that the EU legislations should create uniform access to fertility treatment. They were also more likely to say that all people should be able to use donor gametes. Those who came because they wanted anonymous gametes were more likely to agree that the EU should require anonymity.

Those who wanted gametes not available in their own country were more likely to agree with EU regulations that enabled everyone to be treated and that allowed the use of surrogates. Finally, those who tried in their own countries but were unsuccessful were more likely to agree with a range of attitudes about the EU including that the EU should make it possible to use surrogates, make it quicker, reduce costs, require anonymity and allow people to make their own choice.

As noted, in each case (with two exceptions) even those who felt most strongly about an issue were still less likely than those in Spain to agree with the notion of EU legislation. To pick one example: 42% of those who came to Spain because they wanted anonymous donations agreed that the EU should pass legislation that required anonymity, less than the 71% of those in Spain agreed with that idea. Among those who came because they wanted gametes not allowed in their home country, 63% agreed that the EU should allow for the use of any donor gametes in comparison with 82% of those in Spain (difference not statistically significant).

## Discussion

### Limitations

These data came from an online survey distributed to former patients of a single clinic in Spain. Web surveys generally have relatively low response rates (Couper, 2000; Monroe and Adams, 2012) and this was also true for this survey. On the other hand, concerns about response rates have to be weighed against the advantages online surveys have for trying to reach a generally hard to reach population such as gamete recipients (Freeman et al., 2009). Several additional factors limit the generalizability of our findings. As discussed, patients came to the clinic both as Spanish citizens and as border crossers in search of reproductive care they could not get elsewhere. The border crossers thus represent a number of different countries and their attitudes might be shaped by their nationality as much as by their method of conception (Purewal and van den Akker, 2006, 2007; Schnittker, 2015; Voss, 2000).

### Attitudinal Differences

Our data show a consistent difference in attitudes toward EU legislation between CBRC respondents and respondents from Spain who did not travel across a border for their reproductive care. As others have shown the CBRC clients came to Spain in search of particular kinds of treatment, some of which were not available – or not available to them – in their home countries (Bergmann, 2011; Culley et al., 2011; Gomez and de La Rochebrochard, 2013; Baccino et al., 2014; Kroløkke, 2014). Spain offered treatment to a broad range of clients and appeared to do so more quickly and more successfully than the clients had found in their home countries. For some respondents, an interest in having an anonymous donor drove the border crossing. The respondents from Spain were more interested in seeing the EU adopt legislation that would ease restrictions on access to donor gametes and would make it easier and less expensive for people to be served. In short, they would have liked to see the EU put into place throughout its domain policies similar to those that now prevail in Spain. The CBRC respondents, on the other hand, while they had availed themselves of the existence of such legislation in Spain – and in some cases came to Spain precisely because Spain had fewer restrictions – were more hesitant about creating uniform regulations through the EU itself. This difference between the two sets of respondents remained under a variety of conditions.

In his discussion of the political effects of CBRC, Storrow (2010) noted that CBRC could “temper resistance to restrictions on legislation” because it acts as a kind of “safety-valve” for individuals who might campaign vigorously for reform in their home countries if they were prohibited from traveling elsewhere to find that procedure or treatment. By way of analogy, in the US where anti-abortion laws now limit procedures and stigma permeates the experience of this health service for both patients and providers (O’Donnell et al., 2011; Weitz, 2009), the existence of available abortions in states adjacent to those in which access to abortion is more restrictive might reduce some of the pressure from individuals who can afford to travel towards changing those restrictive laws. Our data cannot confirm or disconfirm Storrow’s hypothesis about the overall effect of CBRC on moral pluralism, especially because we have data only on attitudes at one point in time. However, our findings do suggest that individuals who have crossed borders to receive types of reproductive care not available in their own country do not want to see that kind of care regulated by the EU as much as do those who have not crossed borders. It might be that the border crossers worry that should the debate be taken back to their own country (by way of EU legislation), more rather than less restrictive policies would be put into place and the option they had taken advantage of in the clinic in Spain would be lost altogether. They appear to be more eager to preserve that option – for themselves, for other individuals interested in one of those procedures – than they are in ensuring that that option becomes available elsewhere through EU legislation.

Within the broad range of policies about which we asked questions, different variations of this general argument might apply. Take for example, the issue of requiring anonymity. Only a quarter of all CBRC respondents thought the EU should legislate in that direction whereas 71% of those in Spain – where anonymity is the current law – thought the EU should make this universal. Anonymity is an important reason for those coming from England, Northern Ireland, the Netherlands, and (perhaps) the parts of Australia where anonymity is not allowed. And those who do travel for this reason are more inclined to say that the EU should require anonymity as a blanket policy than are those who did not travel for this reason. However, they are still less likely than the Spain respondents to say that they think this should be EU policy. Even though they see themselves as politically liberal, they oppose their country’s decision to ban anonymity, a decision which has led to national registries and increased involvement of government. (See for the mirror debate in the U.S. both Ertman (2015) and Cohen (2012) who oppose forcing donors to all become open and Cahn (2013) who argues against anonymity.) Knowing how contentious such debates can be, and having observed anonymity lose in their own country, they might be worried that anonymity would disappear altogether rather than be preserved in some enclaves, like Spain. For them, travel across the channel is a small price to pay to preserve a strategy that they find meaningful and significant.

At the other extreme in terms of agreement, half of the CBRC respondents and almost all of the Spain respondents agreed that everyone should be able to be treated for infertility, thus supporting the freedom of movement to legally travel within the EU. Here again, the same pattern prevails: those who came because they were denied treatment in their own country are more inclined than those who came for some other reason to believe that the EU should push for broadened access in all EU countries. But they are not as strongly in favor of this as are the Spain respondents who live in a climate where that access is available to all. As in the case of anonymity, those who have benefited from the varied EU legislation might worry that debates in their home countries about whether older women, single women, lesbians and gay men should have access to reproductive services would result in a situation where more groups were restricted and the option they found would be available to no one without even greater travel.

Alternatively, stemming from the perspective offered by Van Beers (2015), we might imagine that at some level the CBRC respondents accept the symbolic meaning of the laws in their own countries but do not want them to apply in their particular situation, even if they think the laws might be appropriate for others.

A number of voices have been raised against CBRC because of its effects on the population in the country to which people travel. Storrow (2010) mentions the concern of the “exploitation of young gamete providers” and the possible “distortion in the delivery of medical care to the local population,” the latter concern being about costs, waiting times, and priorities. We can read our data from this perspective as well – that those in Spain want EU regulations because they want the services in their own country to be preserved for Spanish citizens and not to be “distorted” by the demand for services by those from other countries. We also observed that almost no one – whether CBRC or not – said that they used the clinic because other places were more expensive. This finding suggests that the particular clinic under consideration is chosen for other reasons and not because it offers its services at a lower cost than other clinics.

Storrow’s (2010) other concern – the exploitation of young gamete providers – is even more difficult to answer with our data. We have no way of knowing the degree to which our Spanish population are aware of the provision of gametes by men and women and whether they see that as a potential problem.

More broadly, the political and legal integration of the EU into a single European market made already existing trade easier while creating new economic opportunities (Fligstein, 1996). The creation of the European Union also opened up new opportunities for European citizens to travel, learn second languages and live in other countries. Fligstein (2008) also looks at changing European identities. He finds that around 13% of the people in Europe think of their European identity as most important. They are the better educated, professional and politically liberal individuals. About half think of themselves as European sometimes. The issue of identity has relevance for a variety of political issues. Fligstein argues that integration on a political issue occurs when the majority of citizens support more integration. Since most citizens retain their country’s identity as primary, political issues remain at the national level (Fligstein 2008). Fligstein does not address CBRC although his concerns about politics and identity are similar to those of Van Beers (2014).

However, extending his arguments provides us with another reason to hypothesize why there has been no consensus or concerted push to create universal regulations concerning reproductive care, even if internal bodies such as ESHRE have made some recommendations in this direction. It remains an empirical question how national beliefs about families create cultural divides about a variety of issues. Just how much information about donors can be made available without protecting the anonymity of donors is a rich debate within EU legislative bodies. These deep-seated disagreements make it all the more unlikely that a uniform set of legal regulations will emerge in the EU with respect to accessibility and information about donor conception.
